# Electrochemical Determination of Food Preservative Nitrite with Gold Nanoparticles/*p*-Aminothiophenol-Modified Gold Electrode

**DOI:** 10.3390/ijms17081253

**Published:** 2016-08-02

**Authors:** Ayşem Üzer, Şener Sağlam, Ziya Can, Erol Erçağ, Reşat Apak

**Affiliations:** 1 Analytical Chemistry Division, Chemistry Department, Faculty of Engineering, Istanbul University, Avcilar, 34320 Istanbul, Turkey; auzer@istanbul.edu.tr (A.Ü.); sener.saglam@istanbul.edu.tr (Ş.S.); ziya.can@istanbul.edu.tr (Z.C.); ercag@istanbul.edu.tr (E.E.); 2 Turkish Academy of Sciences (TUBA) Piyade st. No: 27, 06690 Çankaya Ankara, Turkey

**Keywords:** nitrite determination, gold electrode, modified electrode, gold nanoparticles, square wave voltammetry (SWV)

## Abstract

Due to the negative impact of nitrate and nitrite on human health, their presence exceeding acceptable levels is not desired in foodstuffs. Thus, nitrite determination at low concentrations is a major challenge in electroanalytical chemistry, which can be achieved by fast, cheap, and safe electrochemical sensors. In this work, the working electrode (Au) was functionalized with *p*-aminothiophenol (*p*-ATP) and modified with gold nanoparticles (Au-NPs) to manufacture the final (Au/*p*-ATP-Au_nano_) electrode in a two-step procedure. In the first step, *p*-ATP was electropolymerized on the electrode surface to obtain a polyaminothiophenol (PATP) coating. In the second step, Au/*p*-ATP-Au_nano_ working electrode was prepared by coating the surface with the use of HAuCl_4_ solution and cyclic voltammetry. Determination of aqueous nitrite samples was performed with the proposed electrode (Au/*p*-ATP-Au_nano_) using square wave voltammetry (SWV) in pH 4 buffer medium. Characteristic peak potential of nitrite samples was 0.76 V, and linear calibration curves of current intensity versus concentration was linear in the range of 0.5–50 mg·L^−1^ nitrite with a limit of detection (LOD) of 0.12 mg·L^−1^. Alternatively, nitrite in sausage samples could be colorimetrically determined with high sensitivity by means of *p*-ATP‒modified gold nanoparticles (AuNPs) and naphthylethylene diamine as coupling agents for azo-dye formation due to enhanced charge-transfer interactions with the AuNPs surface. The slopes of the calibration lines in pure NO_2_^−^ solution and in sausage sample solution, to which different concentrations of NO_2_^−^ standards were added, were not significantly different from each other, confirming the robustness and interference tolerance of the method. The proposed voltammetric sensing method was validated against the colorimetric nanosensing method in sausage samples.

## 1. Introduction

Nitrite is an approved additive to meat products as an antimicrobial flavoring and coloring agent, in addition to its ability to retard lipid peroxidation [1]. Another interesting property of nitrite is its capability to retard the development of rancidity during storage, to reduce the thiobarbituric acid (TBA) response (used as a measure of biooxidative status), and the subsequent warmed-over flavors (WOF) developed upon heating of meat [2,3]. Unfortunately, nitrite is an essential precursor in the formation of nitrosamines as a cancer-suspect class of compounds [4]. In the last twenty years, many procedures have been designed to enable sensitive and selective estimation and monitoring of nitrite and nitrate [5], thereby focusing interest on related quantification techniques, such as chromatography [6], spectroscopy [7], and capillary electrophoresis [8]. The first, but still effective, spectrophotometric determination of NO_2_^−^ involved the Griess method, which is more than a century old [9]. Spectrofluorimetry is also a sensitive technique for trace analysis, based on diazotization of nitrite with different reagents [10,11,12]. It should be added that these methods have certain drawbacks, such as toxicity of reagents, tedious extraction procedures, and inherent interference. Electrochemical methods are often favored over others due to their simplicity, rapidity, measurement precision in turbid solutions, and low cost [13,14]. In principle, electrochemical determination of nitrite may involve both oxidation and reduction, but in actual practice, oxidation is usually preferred over reduction because cathodic nitrite measurement is prone to interference from other readily reducible species such as nitrate ion and molecular oxygen [15]. Nitrite is electroactive at traditional electrodes made up of glassy carbon (GC), platinum, diamond, gold, copper, and transition metal oxides [14,16,17,18,19,20,21]. The electrochemical oxidation of nitrite on bare electrodes may be adversely affected by several species (such as nitrite oxidation products and intermediates) which can inhibit electrode processes via irreversible adsorption on the GCE surface and, thereby, decrease both sensitivity and accuracy [18,19,22,23]. In addition, the direct electro-reduction/oxidation of nitrite ions requires high overpotentials at bare electrode surfaces. As a remedy to overcome these limitations, various modified electrodes have been exploited for nitrite determination [24,25,26]. For the purpose of sensing nitrite, electrodes have been chemically modified with facile electron-transfer materials, such as metallophthalocyanines and metalloporphyrins [14,22,27,28,29,30,31,32], inorganic porous substances [33], and enzyme-based electrochemical transducers [34]. In recent years, an increasing variety of nano-materials has been employed for electrochemical studies, combining the advantages of electrochemistry and nanotechnology [35,36,37,38].

Generally compared to a bulk gold electrode, AuNPs-polymer nanocomposites forming an electrocatalytic layer on the surface of a modified electrode are expected to decrease the overpotential and enable faster electron-transfer kinetics for nitrite oxidation [39]. The conducting polymers used for electrode modification mainly comprise polyaniline, polypyrrole, and polythiophene. 4-aminothiophenol (*p*-ATP) is a focusing target in the manufacture of 2D or 3D nanoparticle assemblies by exploiting either covalent or electrostatic (ionic) interactions [40]. Since thiol and amine ends of *p*-ATP have different reactivities [41,42], the effective use of this molecular assembly may give rise to unique morphologies leading to multi-purpose chemical strategies. Moreover, the aromatic (conjugated π-electron system) ring of *p*-ATP intensifies the electrical coupling between NPs and the electrode.

This work reports the fabrication, characterization, and analytical performance of a nitrite sensor involving the incorporation of AuNPs into a conductive polymer matrix, poly(4-aminothiophenol) (PATP), over the surface of a gold electrode. The proposed sensing procedure was tested for the tolerance and elimination of various interferences, and applied to nitrite determination in a real food sample, such as sausage. The nitrite findings obtained with this voltammetric sensor were compared to those found with an AuNPs-based colorimetric sensor also prepared in our laboratory. 

## 2. Results

### 2.1. Fabrication of 4-ATP Polymer Film on an Au Electrode

Electrochemical polymerization of an Au working electrode was achieved in two steps; the –SH groups of 4-ATP were arranged (via self-assembly) on the Au electrode surface, followed by electrochemical polymerization of the *p*-ATP monolayer from the –NH group end with the aid of the cyclic voltammetry (CV) technique [43]. 

**Figure 1:**
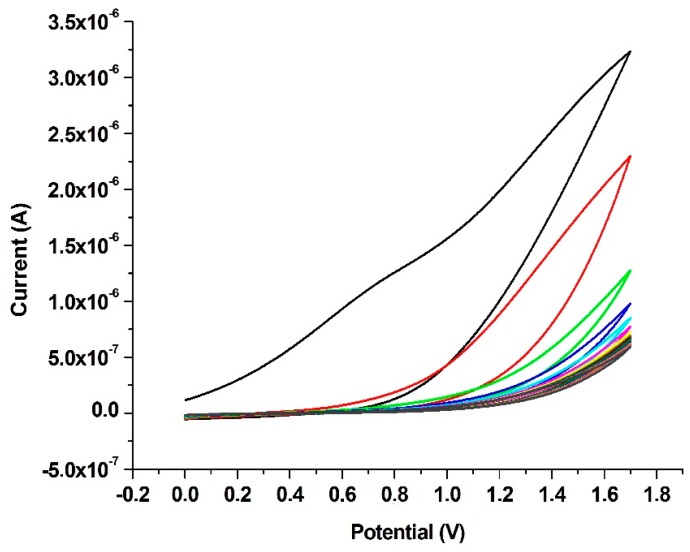
Cyclic voltammograms of –SH groups array on the Au electrode surface (obtained with the use of 5 mL of 10 mM 4-aminothiophenol (*p*-ATP) solution); cycles in numerical order, from top to bottom.

The Au working electrode surface was coated with 10 mM *p*-ATP via CV within the potential range of 0–1.7 V at 20 mV·s^−1^ scanning speed for 20 cycles (Figure 1). As shown in Figure 1, the deposition amount of *p-*ATP on the Au electrode surface increased and, consequently, the current intensities of *p*-ATP (in solution) decreased with an increasing number of cycles (the optimal value of which was set at 20 cycles). As an alternative, this target could be achieved by immersing the working electrode in *p-*ATP solution (physical binding) for 12 h at room temperature. However, if the working electrode is coated electrochemically, it can be used repeatedly. A signal attenuation of about 2% was observed using the working electrode for 15 days, while 80% of the original signal remained after 30 days.

**Figure 2:**
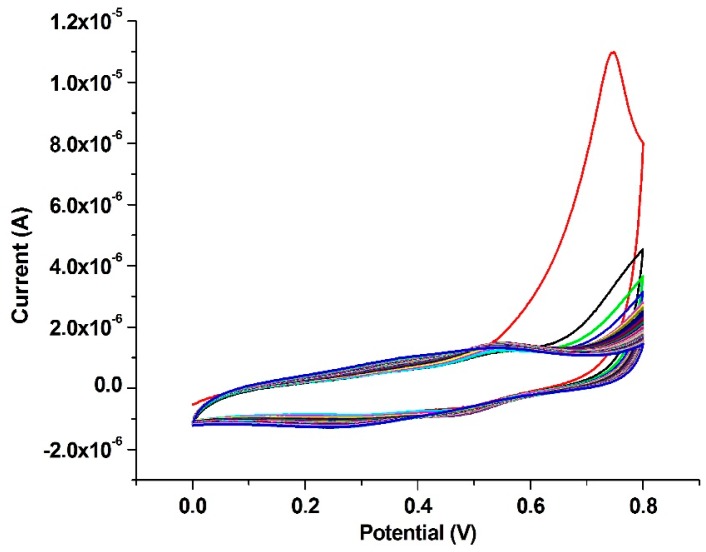
Cyclic voltammograms of electrochemical polymerization monolayer ATP; cycles in numerical order from top to bottom.

At the second step, the Au working electrode surface was immersed in 5 mL of 0.5 M HClO_4_ solution and polymerized via cyclic voltammetry within the potential range of 0–0.8 V at 50 mV·s^−1^ scanning speed for 50 cycles (Figure 2). As a result, the Au working electrode surface was coated with polyaminothiophenol (PATP).

The peak currents centering around 0.7 V were relatively stable for up to 50 voltammetric scans, beyond which a decreasing trend was observed in current. Since the peaks were recoverable in a fresh solution of perchloric acid and similar peaks were not observable with bare gold electrodes, the 0.7 V peak could be attributed to the presence of an electroactive monolayer of poly(4-aminothiophenol) on gold electrode. As the same electroactive film could not be formed in a solution of ATP and ATP→PATP conversion required the preliminary formation of a self-assembled layer on the flat surface of Au, the mentioned peaks were assumed to arise from the formation of polaronic and bipolaronic structures of PATP [44].

### 2.2. Electrodeposition of Au-Nanoparticles on a PATP-Coated Au Electrode

**Figure 3:**
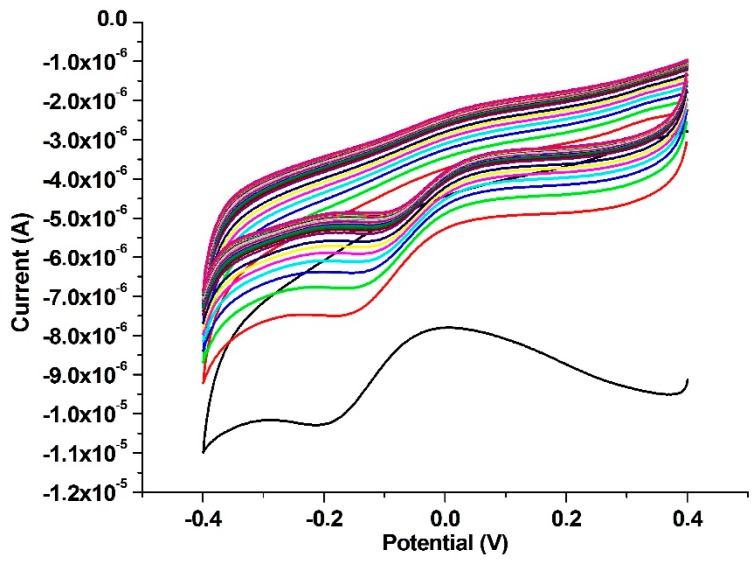
Voltammograms regarding Au nanoparticles on a polyaminothiophenol (PATP) polymer-coated Au electrode; cycles in numerical order, from bottom to top.

The peak manifesting itself at ≈−0.2 V in the first cycle (Figure 3) may be attributed to the reduction of gold nanoparticles on the polyaminothiophenol−coated electrode surface. As the number of cycles was increased, increasing amounts of metallic gold accumulated on the electrode, leaving a smaller number of free Au-NPs (and also diffused Au(III) ions in close proximity to the electrode), leading to a decrease in current intensities in the cathodic range. The current intensity tended to stabilize after 40 cycles, hinting to a saturation of Au-NPs accumulation on the electrode surface. After each regeneration step, material balance regarding gold accumulation on the electrode surface should be made by consideration of the values pertaining to initial and final cycles. An important advantage of this electrode design was time/labor saving by refraining from electrode recoating and cleansing prior to each measurement, as the once prepared Au/PATP-Au_nano_ electrode could be used successively without tedious operations throughout the day.

### 2.3. CV Characterization of the Modified Electrodes

The modified electrodes were characterized by a combination of CV scans, impedance spectral measurements, and scanning electron microscope (SEM) images. For the purpose of electrode characterization, CV was applied in monomer-free medium to bare Au, Au/PATP, and Au/PATP-Au_nano_ electrodes with a scanning speed of 100 mV/s within the potential range of 0–0.8 V.

**Figure 4:**
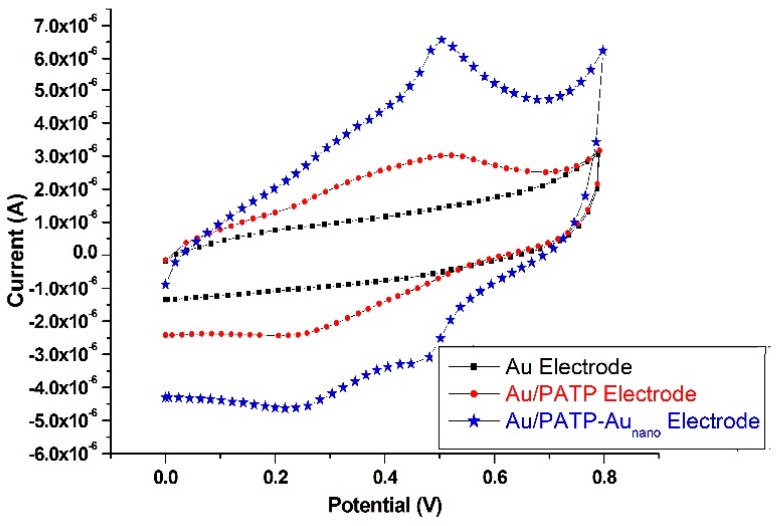
Cyclic voltammetry (CV) characterization of Au, Au/PATP, and Au/PATP-Au_nano_ electrodes.

As can be seen from Figure 4, anodic and cathodic currents could be obtained from both Au/PATP, and Au/PATP-Au_nano_ electrodes, showing that both electrodes have electroactivity and that Au_nano_ coating of the copolymer electrode did not cause a decrease in this activity. For the Au/PATP electrode, anodic and cathodic peaks emerged at 517.9 mV and 214.1 mV, respectively, whereas for the Au/PATP-Au_nano_ electrode, the corresponding peaks shifted to slightly smaller potentials, appearing at 502.6 mV and 225.6 mV, respectively. These lower potential shifts and intensification of currents may be attributed to the increase in effective surface area of the modified electrode [23]. The bare Au electrode did not yield a noticeable peak under identical conditions. 

### 2.4. SEM Images of an Au/PATP-Au_nano_ Electrode

**Figure 5:**
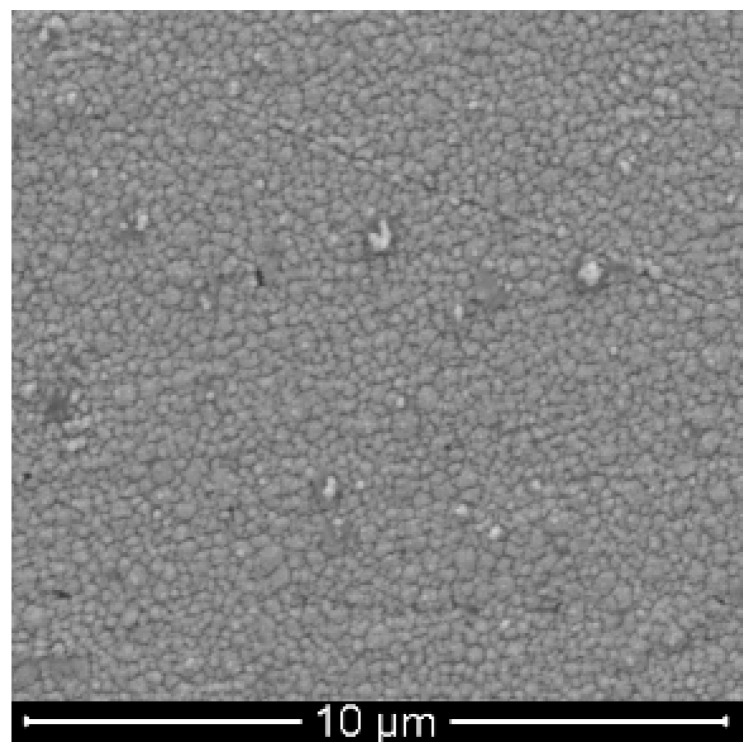
The scanning electron microscope (SEM) images of Au colloidal particles ranging between 75 and 300 nm size (10,000-fold zoom).

The SEM image of the Au/PATP-Au_nano_ electrode was recorded with the aid of a scanning electron microscopy (SEM; FEI Model Quanta 450 FEG, Hillsboro, OR, USA). The SEM images of the produced Au-NPs were shown in Figure 5.

Au-NPs were homogeneously distributed on the *p*-ATP/Au surface with average diameters of about 75 nm; but in addition to well-distributed particles, large aggregated irregular particles with a size of about 300 nm were also observed. Hypothetically, Au-S covalent binding can contribute to Au-NPs immobilization on the electrode surface. This nanostructured film could significantly enhance the active surface area of Au electrode and might be very important to promote electron transfer. Consequently, the resulting modified electrode exhibited a good electrocatalytic capability towards the oxidation of nitrite. 

### 2.5. Electrochemical Impedance Spectroscopy (EIS) Applied to Modified Electrodes

Impedance measurements were made for the Au/PATP and Au/PATP-Au_nano_ electrodes in monomer-free solution media via the potentiostat EIS method; the frequency range was 10 mHz–1 MHz, and points/decade 10 mV. 

**Figure 6:**
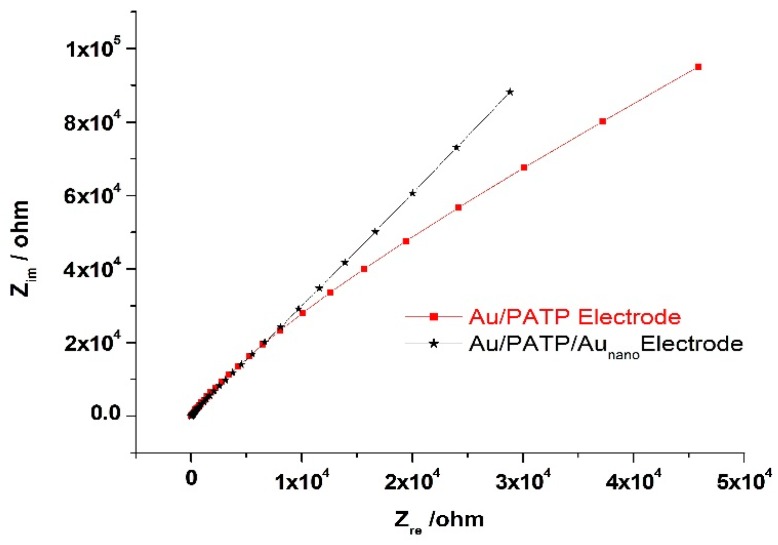
Impedance measurements on the Au/PATP and Au/PATP-Au_nano_ electrodes using the potentiostat electrochemical impedance spectroscopy (EIS) method in monomer-free solution media.

Figure 6 shows the impedance spectra of Au/PATP- and Au/PATP-Au_nano_-modified electrodes. The high frequency region on the left hand side (beginning) of the spectrum identifies the electrolyte properties, while the mid-frequency region corresponds to the electrode/electrolyte interface process. The relaxation effect is represented by a semicircle whose intersections with the real axis are the electrolyte and the charge transfer resistances. Within the region of low frequency, impedance was controlled by counter-ion diffusion inside the electrode; the impedance response, a 45° straight line (Warburg impedance) [45]. The radius of the semicircle for Au/PATP was observed to be smaller than the corresponding value for the Au/PATP-Au_nano_ electrode, indicating a lower charge-transfer resistance (R_CT_) and a higher electroactivity of the former. This may have resulted from the higher thickness of Au/PATP-Au_nano_ electrode. It was reported in other sources that impedance curves depicted on the same Z’/Z” graph enabled the comparison of the electron-transfer capabilities of different (bare and modified) electrodes [46], where the monolayer coated on the modified electrode possibly showed a higher obstruction to electron transfer than the bare electrode [47]. 

In order to gain preliminary information about the charge storage capacity of the electrodes, lower frequency capacitance values (Csp) were calculated as suggested in the literature [48] and found as 1.81 × 10^−4^ and 1.68 × 10^−4^ farad/cm^2^ for Au/PATP and Au/PATP-Au_nano_, respectively, hinting to the better suitability of Au/PATP electrodes for capacitor application.

It should be remembered that while impedance spectral curves enable a comparison of R_CT_ values with slightly increased values of R_CT_ upon Au-NPs modification of the electrode contrary to expectation, the advantages of the Au/PATP-Au_nano_ electrode over Au/PATP have been demonstrated in this work as stability, sensitivity, reproducibility, and repeated use without a need for coating before each application. While studying the electrochemical characteristics of gold electrodes functionalized by carboxyl-terminated alkane thiol monolayers, Bradbury et al. [49] saw that the charge-transfer resistance of the ferri-/ferro-cyanide redox pair at equilibrium potential showed an exponential increase with increasing –CH_2_– units in the monolayer. These authors further argued that adsorption of the citrate-stabilized Au nanoparticles generated a local concentration polarization of the redox species at the interface, leading to an increase of R_CT_. Their preliminary investigations had shown that the apparent charge-transfer resistance in the presence of the Au nanoparticles (R_array_) was strongly dependent on the particle number density [49].

### 2.6. Square Wave Voltammetric Response of the Recommended Sensor Electrode to NO_2_^−^

**Figure 7:**
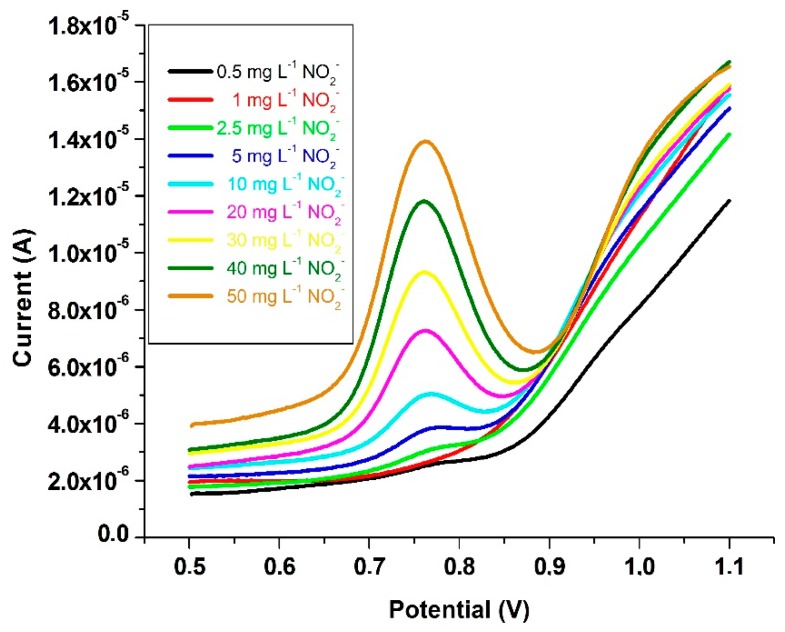
Square wave voltammograms of NO_2_^−^ with Au/PATP-Au_nano_ electrode within the concentrations of 0.5−50 mg·L^−1^ for NO_2_^−^.

Nitrite calibration was done with square wave voltammetry and the characteristic peak potential of NO_2_^−^ appeared at 0.76 V. Square wave voltammograms of NO_2_^−^ recorded within the range of 0.5 and 50 mg·L^−1^ (final concentrations) were given in Figure 7. Calibration of nitrite at 0.76 V potential gave a linear dependence of current intensity versus concentration:

I_0.76 V_ = 0.234 C_NO_2__^−^ + 2.537 (*r* = 0.999) (1)

where I_0.76 V_ is the peak current intensity (µA) at 0.76 V and C_NO_2__^−^ is the NO_2_^−^ concentration (mg·L^−1^).

The calibration curve for the analr Nyte is established as response (*y*) versus concentration (*x*). The minimal analyte response that can be detected (*y*_LOD_) corresponding to the concentration at the limit of detection (*x*_LOD_) is equal to the mean value of blank responses (*ӯ*_bl_) exceeded by (*ks*_bl_), where s_bl_ is the standard deviation of blank responses and *k* is a factor equal to 3 (by International Union of Pure and Applied Chemistry (IUPAC) recommendation) such that *y*_LOD_ = *ӯ*_bl_ + *ks*_bl_ = *ӯ*_bl_ + 3*s*_bl_. Thus, the calibration curve is intersected at *y*_LOD_ response value, a vertical line is drawn from this intersection up to the horizontal axis, and the corresponding concentration (*x*_LOD_) is directly read on the intersection point of this perpendicular line with the horizontal axis [50]. In this work, LOD was assessed by calculating the peak current intensity at three standard deviations above the mean current intensity after *n* = 10 repetitive measurements of a reagent blank not containing any analyte (nitrite).

The same procedure at ten standard deviations above the mean of the blank intensity was performed for the limit of quantification (LOQ) calculation. LOD and LOQ values of analytical results were found to be 0.12 and 0.40 mg·L^−1^, respectively. Thus, the developed sensor electrode was capable of detecting very low concentrations of nitrite, which may be important for food analysis. The analytical performance of the developed sensor was compared with those of other electrochemical methods utilizing nanoparticle-based sensor electrodes (Table 1). The reproducibility of the voltammetric sensor (Au/*p*-ATP-Au_nano_) for nitrite determination was investigated with intra- and inter-assay precision measurements. 

The intra-assay precision of the sensor was evaluated by determining 10 mg·L^−1^ nitrite with the same Au/*p*-ATP-Au_nano_ electrode after five successive determinations. The inter-assay precision, or the fabrication reproducibility, was estimated by detecting the same amount of nitrite in duplicate with five sensor electrodes prepared in the same manner independently. The resulting intra- and inter-assay precisions in terms of percentage Relative Standard Deviation (RSD) values were 3.74% and 8.54%, respectively, indicating high reproducibility. In addition, there was no significant decrease in current response in the detection of 10 mg·L^−1^ nitrite during the first seven days. 

### 2.7. Colorimetric Sensor Response to NO_2_^−^

A colorimetric sensor was applied to the determination of nitrite yielding the calibration curve:

A_565 nm_ = 0.1331C_NO_2__^−^ − 0.0508 (*r* = 0.9992) (2)

where C_NO_2__^−^ is the NO_2_^−^ concentration (in mg·L^−1^) in the final solution and the molar absorptivity is ε = 6.52 × 10^3^ L·mol^−1^·cm^−1^ with a limit of detection (LOD) = 0.23 mg·L^−1^ and a limit of quantification (LOQ) = 0.76 mg·L^−1^ (*LOD* = 3*σ*_bl_/*m* and *LOQ* = 10*σ*_bl_/*m*, where σ_bl_ denotes the standard deviation of a blank and *m* is the slope of the calibration curve for spectrophotometric nitrite determination).

**Figure 8:**
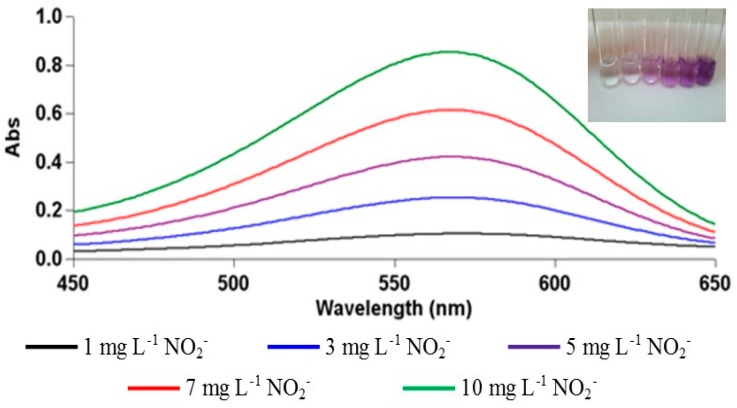
Spectra of NO_2_^−^ with respect to the colorimetric sensing method within the initial concentration range of 1−10 mg·L^−1^. The color images of the test tubes containing from left to right, blank sample and colors obtained from samples of 1–10 mg·L^−1^ concentration range are shown in the inset figure.

The spectra for nitrite determination at different concentrations are shown in Figure 8.

### 2.8. Real Sample Analysis

**Figure 9:**
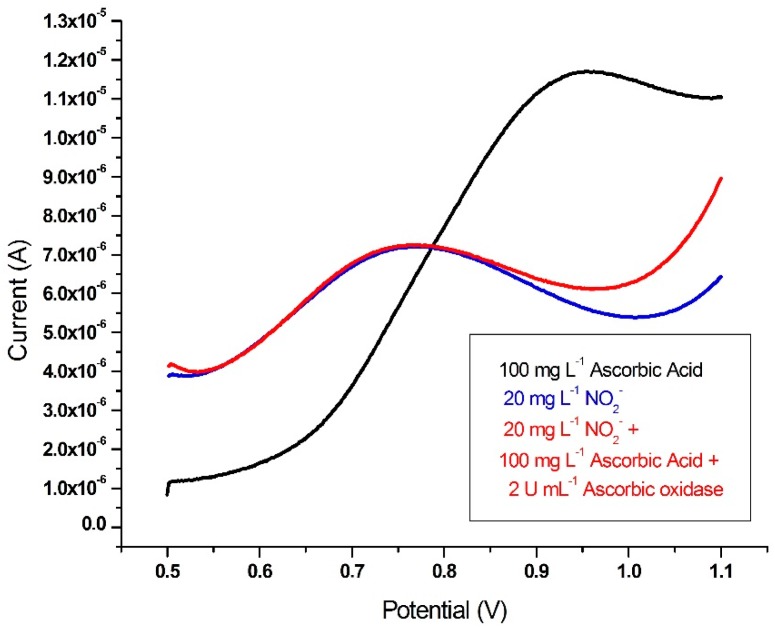
Square wave voltammograms of 20 mg·L^−1^ NO_2_^−^, 100 mg·L^−1^ ascorbic acid and the mixture of 20 mg·L^−1^ NO_2_^−^ + 100 mg·L^−1^ ascorbic acid + 100 mg·L^−1^ ascorbic acid oxidase.

***Analysis of NO_2_^−^ in sausage samples after preliminary extraction with the CUPRAC reagent*:** When nitrite amount of sausages samples was measured with the proposed method (SWV), an interference effect was observed owing to the reducing constituents and additives in these samples. Firstly the interference effect of ascorbic acid (AA), which is added to sausage samples as an antioxidant to protect from light and to stabilize the color, was tested and eliminated. For this purpose, 100 mg·L^−1^ ascorbic acid was measured with SWV and characteristic peak potential appeared at 0.97 V. This potential was close to the characteristic peak potential of NO_2_^−^ (at 0.76 V). Therefore, ascorbic acid oxidase was used to inhibit the ascorbic acid-originated interference. The synthetic solutions of 20 mg·L^−1^ NO_2_^−^, 100 mg·L^−1^ ascorbic acid and the mixture of 20 mg·L^−1^ NO_2_^−^ + 100 mg·L^−1^ ascorbic acid + 2 U·mL^−1^ ascorbic acid oxidase (in final solution) were separately measured with the proposed method. As shown in Figure 9, the interference of AA was eliminated with ascorbic acid oxidase (AAO) as the analyte-specific enzyme responsible for its oxidation, and the recovery was found to be 100.5% for NO_2_^−^.

Although AA interference could be overcome with the AAO enzyme, other electro-active food additives of sausage still interfered with nitrite determination. In order to remove all reducing agents having possible interferent effects, a pre-extraction procedure associated with the modified CUPRAC method was applied. In this method, pre-prepared sausages were extracted with Cu(II)-neocuproine into dichloromethane, the reducing constituents oxidized with the CUPRAC reagent and the resulting yellow-orange colored organic phase discarded, and finally, the developed electrochemical method applied to NO_2_^−^ estimation in sausage samples. Nitrite remained unaffected by the modified CUPRAC extraction. 

The standard potential for the nitrate-nitrous acid reduction reaction is given as [55]:

(3) $$\begin{array}{c} {{\text{NO}}_{3}}^{-}+3H^{+}+2e^{-}↔{\text{HNO}}_{2}+H_{2}O\\ E^{o}=+0.94\;V \end{array}$$

Therefore, nitrite—having such a high potential—behaves as a reducing agent only for strong oxidants (like permanganate, having a redox potential of +1.51 V in 1.0 N sulfuric acid medium) in acidic pH, for example:

5 NO_2_^−^ + 2 MnO_4_^−^ + 6 H^+^ → 5 NO_3_^−^ + 2 Mn^2+^ + 3 H_2_O (4)

On the other hand, Cu(II)-neocuproine complex has a redox potential of about +0.60 V at neutral pH. Therefore, thermodynamically speaking, Cu(II)-neocuproine cannot oxidize nitrite to nitrate, and is itself reduced to the highly-colored Cu(I)-neocuproine chelate at neutral pH. Tütem et al. [56] showed that Cu(II)-Nc does not oxidize nitrite (and is itself reduced to a colored product) at the working pH of the method. 

Table 2 shows the results obtained by simultaneously applying the electrochemical and colorimetric sensors to sausages samples, prepared as described in “Materials and Methods”.

Statistical comparison between the results of the proposed voltammetric and reference colorimetric sensor procedures applied to sausage sample (for Brand “B”) was made on *n* = 5 repetitive determinations, essentially not showing significant differences between the results (Table 3). 

The confidence level used in method validation for sausage sample was 99% and 95%, respectively, for *t*- and *F*-tests.

In the literature, noble metal nanoparticle-based electrochemical sensors have usually been tested in real matrices, such as sausage, milk, table salt, and tap water, while sausage is the most frequently utilized and most complex sample for nitrite. Nitrate and nitrite are used in dry-fermented sausage manufacture [57], for which comparisons are frequently made between samples manufactured with nitrite and those processed incorporating both nitrite and nitrate [58,59]. According to the Turkish Food Codex (TFC-Regulation) issued in the Republic of Turkey Official Newspaper, with Number 28693 and date 30 June 2013, nitrite is a permissible food additive that can be added to certain meat products, including sausage, while nitrite cannot be added to milk products. The nitrite contents of brand B and brand C sausages were below the permissible levels (60 ppm) with respect to the TFC-Regulation, while brand A exceeded this limit (Table 2).

Most electrochemical sensors published previously have not considered the analytical problem of interference elimination while determining nitrite in sausage samples. In a study of Yang et al. [60], a phenoxazine dye was electropolymerized on GCE, and the manufactured amperometric sensor was used in the anodic oxidation of nitrite, but ascorbate was observed to seriously interfere (i.e., with a signal change >30% for one-fold molar ratio of ascorbate), and no measure could be taken to eliminate this interference. Saber-Tehrani et al. [40], using a Pt-NPs distributed poly(2-aminothiophenol)-modified electrode, reported that ascorbate, at a five-fold mass ratio to nitrite, caused a current attenuation exceeding 10%, while Wang et al. [61] noted a signal change >5% at 20-fold ascorbate with the use of a Au-NPs on choline chloride modified GCE. Cui et al. [24] used a chitosan-coated Prussian blue nanoparticle sensor electrode in nitrite determination, without mentioning how ascorbate interference was removed. In comparison to three of the methods listed in Table 1, the proposed method had a lower LOD of 2.6 µM. The electrode prepared by Miao et al. [52] used the corrosive and hazardous acid mixture called ‘piranha solution’ for cleansing, and electrode manufacture was cumbersome (requiring heat treatment at 95 °C for 20 min). Most of the listed methods did not report how interferences (arising from ascorbic acid and some amino acid residues on proteins) were eliminated. 

## 3. Discussion

This study provided a novel *p*-aminothiophenol (*p*-ATP)-modified and gold nanoparticles-derivatized gold electrode (Au/*p*-ATP-Au_nano_) for nitrite determination in food samples. The measurements were conducted in pH 4 buffer medium due to the decrease of current intensities below pK_a_ of HNO_2_ (pK_a_ between 3.2 and 3.4) [23] arising from the decomposition of free nitrous acid into N-oxides (2 HNO_2_ → NO + NO_2_ + H_2_O). Electrochemical approaches are favorable for nitrite determination owing to rapid response, simple operation, and capability of measurement in turbid samples. The developed sensor had good performance in food samples, and was sensitive and stable. The advantage of the co-deposition modification may find further utilization in the field of electrochemical sensing. Compared to the conventional GC electrode showing an oxidation peak at ca. +0.8 V for nitrite, this modified nano-electrode showed a lower potential (at ca. +0.76 V) with intensified current, possibly due to increased effective surface area favorably changing the diffusion regime through the nanoparticles dispersed in the electrode surface film [23]. The repeated use of the electrode without loss of reliability confirmed the absence of surface contamination by irreversible adsorption of nitrite oxidation products and other electrochemical byproducts, and this constitutes a distinct advantage over bare electrodes used for the same purpose. The interference-free utilization of the proposed voltammetric sensing method was also investigated by analysis of nitrite in sausage samples. The slopes of the calibration lines in pure NO_2_^−^ solution and in the sausage sample solution to which different concentrations of NO_2_^−^ standards were added were not significantly different from each other, confirming the robustness and interference tolerance of the method. As a major advantage over colorimetric methods employing the Griess reaction, nitrite can be easily determined with the recommended electrochemical method in admixture with nitrate, as the latter is electrochemically inactive. Generally, there is a lack of sufficient information regarding the interference effects of food additives on determination of nitrite, and the counter measures to compensate for possible interferences were not effectively discussed for various modified electrodes presented in literature. Especially, the adverse effect of ascorbic acid (causing a close potential peak overlap) is undeniable. The proposed method distinguishes itself from similar other methods reported in literature with its effective interference removal technique (i.e., removal of ascorbic acid with ascorbate oxidase, and removal of other chemical reductant interferents with Cu(II)-neocuproine extraction into dichloromethane) prior to electrochemical measurement. The sensitivity and selectivity of the proposed method is sufficient to assess the compatibility of meat products (such as sausage) to TFC-Regulation.

## 4. Materials and Methods

### 4.1. Chemicals, Solutions, and Instruments

The alumina slurry used for electrode cleaning was from Baikowski International Corp (Charlotte, NC, USA) (0.05 µm, Baikalox 0.05CR). The supporting electrolyte for conductivity was 0.1 M pH:4 phosphate buffer solution (NaH_2_PO_4_–Na_2_HPO_4_) (Merck, Darmstadt, Germany). For electrode cleanliness, isopropyl alcohol (Sigma-Aldrich), acetone, and ethanol (both technical grade) were used. *N*-(1-naphtyl)-ethylenediamine dichloride (NED) (used in colorimetric sensor determination) was obtained from Fluka (St. Louis, MO, USA). The electrode coating material, 4-aminothiophenol (4-ATP), as well as the rest of the reagents, were supplied from E. Merck (Darmstadt, Germany). 

Gold(III) chloride solution (99.99% trace metals basis, 30% by wt. in dilute HCl) was used for deposition of gold nanoparticles to Au electrode coated with PATP copolymer, and the 0.04% (*w*/*v*) working solution was prepared from this stock solution. The final chemical form of Au(III) in acidic solution was HAuCl_4_. 

Preparation of Solutions in colorimetric sensor determination of NO_2_^−^: The working solutions of NO_2_^−^ at 1–10 mg·L^−1^ were prepared from the corresponding aqueous stock solution at 500 mg·L^−1^. H_3_PO_4_ (0.1 M) and NED (20 mM) solutions were prepared in pure water and 4-ATP (10 mM) solution in absolute ethanol. 

Voltammetric experiments were performed with a Gamry Instruments model Reference 600 potentiostat/galvanostat/zero resistance ammeter (ZRA) interfaced to a PC computer and controlled Gamry Framework software (Warminster, PA, USA). Gold (BASi stationary voltammetry electrodes; ø 1.6 mm,) was used as the working electrode. A platinum (Pt) electrode and an Ag/AgCl, 3 M KCl electrode served as the auxiliary and reference electrodes, respectively. Optical absorption measurements were carried out in matched Hellma quartz cuvettes using a Varian CARY Bio 100 UV-VIS spectrophotometer (Agilent Technologies, Santa Clara, CA, USA).

### 4.2. Optimization of Voltammetric Method

Electrode type, supporting electrolyte, scan rate, and concentration parameters were individually examined. A gold electrode was selected as the working electrode, by which a substantial signal could be produced (as current intensity) at sufficiently separated reduction potentials. A scan rate of 50 mV·s^−1^ was chosen, and 0.1 M phosphate buffer solution was preferred as the supporting electrolyte. The electrolyte pH was found to be optimal at pH 4 due to the stability problem of nitrite at more acidic pH. 

### 4.3. Gold Electrode Pre-Treatment

The Au electrode was polished with a suspension of alumina powder in the presence of pure water on a smooth polishing cloth [62] by circular movements for a few minutes, then washed with distilled water, and sonicated for 5 min in bi-distilled water. Sonication of the electrode was repeated for another 5 min in isopropyl alcohol–acetonitrile (1:1, *v*/*v*) mixture. The efficiency of this procedure for electrode cleanliness was confirmed from the absence of any baseline (blank) peak during CV scan.

### 4.4. Electrochemical Polymerization of PATP Film

The monomer solution (10 mM 4-ATP) was prepared in ethanol. A three-electrode cell was used; an Au working electrode, a platinum wire counter electrode, and an Ag/AgCl reference electrode. Electrochemical polymerization of PATP film was achieved in two steps. In the first step -SH groups of 4-ATP were arranged by self-assembly on the Au electrode surface. Five milliliters of monomer: 4-aminothiophenol solution was taken into the working cell. Polymerization was performed via cyclic voltammetry within the potential range of 0–1.7 V at 20 mV·s^−1^ scanning speed for 20 cycles. The second step was carried out in a 0.5 M HClO_4_ solution. Electropolymerization monolayer ATP was formed by CV method from the –NH group end. Polymerization was performed via cyclic voltammetry within the potential range of 0 V to 0.8 V at 50 mV·s^−1^ scanning speed for 50 cycles. Finally, the modified Au electrode was rinsed with distilled water to remove any unbound material from the surface.

### 4.5. Electrodeposition of Au Nanoparticles on the Polymer Coated Electrode

PATP/Au electrode was coated with Au nanoparticles using cyclic voltammetry with 0.04% (*w*/*v*) HAuCl_4_ (2.5 mL) + 0.1 M H_2_SO_4_ (2.5 mL) solutions via electrochemical deposition process. Coating was performed within the (−0.4 V to 0.4 V) range at 50 mV·s^−1^ scanning speed. The amount of deposited gold particles onto the PATP/Au surface was determined by checking cycle numbers, the optimal value of which was set at 40 cycles. The golden-colored electrode formed in this way was named as Au/PATP/Au_nano_

### 4.6. Electrochemical Determinaion of NO_2_^−^

The working solutions of 2.5–250 mg·L^−1^ NO_2_^−^ were prepared from the stock solution and transferred to the measurement cell; one milliliter of NO_2_^−^ was added and the cell was filled up to 5 mL with pH:4 phosphate buffer solution (NO_2_^−^ final concentration range was 0.5–50 mg·L^−1^). Square wave voltammetry (SWV) was performed in a potential range from 0.5–1.1 V, step size 2 mV and pulse size 25 mV, equilibrium time 15 s. The analytical experiment was carried out at a frequency of 50 Hz and the characteristic peak potential of NO_2_^−^ was determined.

Square wave voltammetry was performed as follows: the electrode was held at conditioning potential −0.5 V for 5 s, then equilibrated for 2 s; the analytical experiment was carried out at 25 Hz frequency from initial potential −0.5 V to end potential 1.2 V, where the step potential was 0.00195 V and amplitude 0.01245 V.

### 4.7. Preparation of Colorimetric Sensor for NO_2_^−^ Determination

Preparation of 4-ATP Functionalized AuNPs. 

Synthesis and modification of AuNPs was carried out by a previously published method in our laboratory [63]. The methods were originally developed for the determination of nitrite, derived from the hydrolytic cleavage of heterocyclic nitramine energetic materials (i.e., hexahydro-1,3,5-trinitro-1,3,5-triazine (RDX) and octahydro-1,3,5,7-tetranitro-1,3,5,7-tetrazocine (HMX).

Fifty milliliters of 0.002% HAuCl_4_ was heated to boiling. To this solution, 0.5 mL of 1% trisodium citrate was added. The solution was heated until its color changed to the red-wine characteristic of the surface plasmons of gold, then removed from the hot plate, and cooled to room temperature. AuNPs were further functionalized in the same solution (i.e., without centrifugation) with 4-ATP. First, the pH of AuNPs was adjusted to 3.0 where the thiol group of 4-ATP showed maximum affinity toward the surface of AuNPs. This acidic AuNPs solution was mixed with 10 mM 4-ATP at 8:1 (*v*/*v*) ratio and then stirred at 60 °C for 3 h. The solution was then left to stabilize at room temperature for 48 h. After functionalization, 4-ATP−AuNP remained useful for quantitative analysis for at least two days without losing its maximal absorbance for nitrite.

The scheme for the colorimetric sensor is summarized as follows: take 2 mL NO_2_^−^ sample; add 1.0 mL of AuNP-4ATP + 0.2 mL of 0.1 M H_3_PO_4_ + 0.5 mL of 20.0 mM NED in this order; measure A_565 nm_ against a reagent blank after 30 min of NED addition.

### 4.8. Application of Voltammetric and Colorimetric Sensors to Sausage Samples

Three different brands of sausage samples were used for analysis. In accordance with literature [53], 10 g of sausage was shredded, 12.5 mL of saturated borax solution added, and then put into a 70 °C water bath for 15 min. At the end of this time, the proteins in the sausages were precipitated by adding 2.5 mL of 30% ZnSO_4_ solution. After that, the samples were put into a centrifuge tube and centrifuged for 10 min at 8000 rpm and filtered through Glass Fiber/PolyEthylene Terephthalate (GF/PET) 45/25 filter via a syringe, and finally diluted to 50 mL. Both voltammetric and colorimetric sensors were applied to the obtained samples. 

Voltammetric Sensing of NO_2_^−^ in Sausage Samples: Since the direct determination of nitrite in the sausage samples with the developed voltammetric method was not possible due to the oxidation of other interfering constituents at close potentials, a preliminary extraction was performed, based on the CUPRAC method existing in literature [64].

Preliminary treatment and interference removal of sausage samples with an extractive CUPRAC method: 0.5 mL 10 mM CuCl_2_·2H_2_O + 1.2 mL 10 mM Nc + 2 mL pH:7 (NH_4_Ac) buffer + 4 mL sausage sample + 4 mL dicloromethane (after this extraction, 7.3 mL aqueous extract obtained).

Voltammetric measurement on sausage samples after CUPRAC extraction: In the measurement cell; 0.5 mL sample + 0.5 mL water + 4 mL pH:4 buffer. Scan rate: 50 mV·s^−1^, readings were made with SWV within the 0.5–1.1 V potential range.

Colorimetric Sensing of NO_2_^−^ in Sausage Samples: The determination of nitrite in sausage samples with the use of the colorimetric sensor was performed using the method of standard additions without any need of pre-extraction into organic solvent with the CUPRAC reagent.

The method of standard additions was applied to the extracted sausage samples as follows:

Two milliliters of extracted sausage samples + nitrite addition within the linear calibration range; dilute with distilled water to 10 mL. As unspiked sample, 2 mL of sausage sample extract was also taken and diluted to 10 mL.

The previously developed method for the determination of heterocyclic nitramine energetic materials, abbreviated as “4-ATP-AuNP+NED”, was modified for the determination of nitrite and standard-added samples as follows:

Two milliliters of NO_2_^−^ or standard-added samples + 1 mL AuNP-4ATP + 0.2 mL of 0.1 M H_3_PO_4_ + 0.5 mL of 20 mM NED, taken for colorimetric determination.

## Figures and Tables

**Table 1:** The comparison of analytical figures of merit with other electrochemical methods utilizing nanoparticle-based sensor electrodes.

Electrode Material	Method	LOD µM	Linear Range	Reference
**EPPGE/SWCNT/Co and CoO*_x_* NP**	Chronoamperometry	5.6	32.3–189 µM	[51]
**GE/AEBA/DPAN/PtNPs**	Amperometry	5	1–10 µM	[52]
**ACNTs/thionin modified**	Differential Pulse Voltammetry (DPV)	1.12	3 × 10^−6^–5 × 10^−4^ mol·L^−1^	[53]
**GE/PATP-Ptnano**	Cyclic Voltammetry (CV)	1	3 µmol·L^−1^–1 mmol·L^−1^	[40]
**PGCE/nanometer-sized gold colloid/ethylenediamine**	Amperometry	45	1.3 × 10^−4^–4.4 × 10^−2^ mol·L^−1^	[54]
**GE/*p*-ATP-Au_nano_**	Square Wave Voltammetry (SWV)	2.6	0.5–50 mg·L^−1^	Üzer et al. (Proposed method)

LOD: limit of detection; EPPGE: edge plane pyrolytic graphite electrode; GE: gold electrode; SWCNT: single-walled carbon nanotubes; CoO*_x_*: cobalt oxide; ACNTs: carbon nanotubes; AEBA: 4-(2-aminoethyl)benzenamine; DPAN: 5-[1,2]dithiolan-3-yl-pentanoic acid [2-(naphthalene-1-ylamino)-ethyl]amide; PtNPs: platinum nanoparticles; PGCE: pretreated glassy carbon electrode.

**Table 2:** Voltammetric and colorimetric results of three different brand sausages samples (number of measurements for both methods: *n* = 5).

Method	Brand “A”	Brand “B”	Brand “C”
Voltammetric	22.48 mg·L^−1^	5.83 mg·L^−1^	9.47 mg·L^−1^
Colorimetric	17.64 mg·L^−1^	5.18 mg·L^−1^	7.71 mg·L^−1^

**Table 3:** Statistical comparison of the proposed voltammetric sensor with a colorimetric sensor method applied to a sausage sample (number of measurements for both methods: *n* = 5).

Method	Mean Conc. (mg·L^−1^)	SD (σ)	*S* ^a,b^	*t* ^a,b^	*t*_table_^b^	*F* ^b^	*F*_table_^b^
Voltammetric	5.83	0.522	-	-	-	-	-
Colorimetric	5.18	0.276	0.418	2.402	3.355	0.278	6.39

^a^ *S*^2^ = ((*n*_1_ − 1)*s*_1_^2^ + (*n*_2_ − 1)*s*_2_^2^)/(*n*_1_ + *n*_2_ − 2) and *t* = (*ā*_1_ − *ā*_2_)/(*S*(1/*n*_1_ + 1/*n*_2_)^1/2^), where S is the pooled standard deviation, s_1_ and *s*_2_ are the standard deviations of the two populations with sample sizes of n_1_ and n_2_, and sample means of *ā*_1_ and *ā*_2_, respectively (*t* has (*n*_1_ + *n*_2_ − 2) degrees of freedom); here, *n*_1_ = *n*_2_ = 5; ^b^ The statistical comparison is made on paired data produced with proposed and reference methods; the results are given only on the row of the reference method. Conc.: concentration, SD: standard deviation
